# Rhabdomyolysis and Pandemic (H1N1) 2009 Pneumonia in Adult

**DOI:** 10.3201/eid1603.091818

**Published:** 2010-03

**Authors:** Ramiro L. Gutierrez, Michael W. Ellis, Catherine F. Decker

**Affiliations:** National Naval Medical Center, Bethesda, Maryland, USA (R.L. Gutierrez, C.F. Decker); Uniformed Services University of the Health Sciences, Bethesda (M.W. Ellis, C.F. Decker)

**Keywords:** Influenza, pandemic (H1N1) 2009, multiple myeloma, rhabdomyolysis, infection, letter

**To the Editor**: A 56-year-old man came to the emergency department (ED) of Malcolm Grow Medical Center at Andrews Air Force Base in suburban Maryland, USA, just outside Washington, DC. He had a history of several days of cough, fever, and malaise; was a nonsmoker; was overweight (body mass index 28 kg/m^2^); and did not have chronic pulmonary disease. Radiographs showed bilateral pulmonary infiltrates, and he was hypoxemic. Two weeks previously, the patient had begun receiving therapy for recurrent multiple myeloma (lenalidomide and high-dose dexamethasone). He was intubated at the time of initial visit to the ED for influenza symptoms, and broad-spectrum antimicrobial drugs were administered (vancomycin 1,000 mg every 12 h, piperacillin-tazobactam 4.5 gm every 6 h, and levofloxacin 750 mg 1×/d). Initial nasopharyngeal wash was negative for influenza A and B antigen by enzyme immunoassay; serum creatine kinase was 271 U/L (reference range 38–174 U/L).

After oxygenation worsened, bronchoalveolar lavage was performed, and drug therapy was broadened to include voriconazole, trimethoprim-sulfamethoxazole, and prednisone. Bronchoalveolar lavage viral culture was positive for influenza A, and real-time reverse transcription–PCR confirmed pandemic (H1N1) 2009 virus infection. Therapy with oseltamivir (75 mg every 12 h) was initiated, and the patient’s respiratory status gradually improved. On hospital day 14, total creatine kinase levels were elevated at 4,854 U/L and rose to 76,015 U/L over the next 4 days before decreasing. Urine myoglobin peaked at 286,000 μg/L (reference range 0–28 μg/L). Renal function remained at baseline until the patient was discharged 2 weeks later; measured glomerular filtration rates were >120 mL/min. Hydration with normal saline and supportive care was provided; the patient was extubated and discharged to a rehabilitation hospital on hospital day 19. No medications or other treatments could be implicated as the cause of rhabdomyolysis in this patient.

More commonly reported in children, myositis associated with influenza A and B has been well documented and appears to occur most often during the convalescent phase of illness (*1*). Influenza-associated rhabdomyolysis with myoglobinuria have been shown to complicate 3% of cases of myositis in children, are more likely to be associated with influenza A infection (*1*), and have been associated with renal insufficiency requiring renal replacement therapy (*1**,**2*). The frequency of myositis or rhabdomyolysis among adults with pandemic (H1N1) 2009 infection is unclear, but a recently published case series of 18 severely ill patients in Mexico showed that mild to moderate creatine kinase elevation (1,000–5,000 U/L) occurred in >60% of tested patients (*3*). A report from Australia documented rhabdomyolysis as a complication of pandemic (H1N1) 2009 infection in a 16-year-old-boy (*4*), and, more recently, a case of rhabdomyolysis was reported in a 28-year-old patient (*5*).

Our case demonstrates rhabdomyolysis with myoglobinuria that arose during convalescence from severe pandemic (H1N1) 2009 pneumonia in an immunocompromised adult. It is yet to be determined whether pandemic (H1N1) 2009 virus infection has a higher propensity toward muscular inflammation than do other viral infections or seasonal influenza. Rhabdomyolysis should be considered in the evaluation of muscle symptoms associated with pandemic (H1N1) 2009 virus infection, especially among critically ill patients. When influenza suspicion is high, obtaining bronchoalveolar lavage specimens for viral culture, PCR, and antigen testing should be considered if nasopharyngeal sampling and testing for influenza antigen and viral culture are initially negative.
